# Level of sex hormones and their association with acetylsalicylic acid intolerance and nasal polyposis

**DOI:** 10.1371/journal.pone.0243732

**Published:** 2020-12-17

**Authors:** Julia Espersen, Ursula Weber, Ariane Römer-Franz, Thomas Lenarz, Stefan R. O. Stolle, Athanasia Warnecke

**Affiliations:** Department of Otolaryngology, Hannover Medical School, Hannover, Germany; University of Limerick, IRELAND

## Abstract

**Background:**

Chronic rhinosinusitis may be associated with nasal polyposis. Recurrence of disease is often observed and may be due to an intolerance of acetylsalicylic acid. Sex hormones are known to modulate allergic reactions and inflammation. Whether they may be involved in the development and progression of nasal polyposis has not been investigated yet.

**Aim:**

Examine the relationship between levels of sex hormones and nasal polyposis.

**Methods:**

Hormonal levels (estradiol, testosterone and progesterone) in patients with nasal polyposis (n = 26) with or without acetylsalicylic acid-intolerance were determined and compared to hormonal levels in patients with septal deviation (n = 35). Cone-beam computed tomography scans were analysed by using scores as defined by Lund and Mackay and by Kennedy.

**Results:**

Our results show a 5 times greater odds (p = 0.01) for developing nasal polyposis in the presence of lowered estradiol plasma levels than in the presence of normal / elevated levels. When analyzing females and males separately, a 6 times greater odds for females to develop nasal polyposis in the presence of lowered estradiol plasma levels was calculated (p = 0.02). Thus, females are more likely to develop nasal polyposis when they have lowered estradiol levels than males. In addition, female patients showed an increased risk for developing ASA intolerance (p = 0.01).

**Conclusion:**

Variation of sex hormones may be involved in nasal polyposis. Further studies including more patients to validate the presented results are required.

**Significance:**

Retrospective clinical investigation suggesting a correlation between varying sex hormones and nasal polyposis.

## Introduction

More than 10% of the adult population suffers from chronic rhinosinusitis (CRS) and up to 25% of the cases are associated with nasal polyposis [1]. Interestingly, CRS seems to be associated with cognitive dysfunction [2] as shown by cognitive assessment tests, e.g., the Cognitive Failure Questionnaire and the Automated Neuropsychological Assessment Metrics platform [2]. Pro-inflammatory molecules might be the main link between cognitive dysfunction and CRS [2]. A higher level of stress as assumed for patients with CRS and nasal polyposis might influence the severity of cognitive dysfunction negatively [2]. Several studies have shown a correlation between the presence of pro-inflammatory molecules and cognitive dysfunction [3–5]. Existing hypotheses postulate an individual effect of particular inflammatory mediators on cognitive function in CRS with or without nasal polyposis [2]. Referring to this study [2], the inflammatory cascade might impact cognition.

### Chronic rhinosinusitis and aspirin-exacerbated respiratory disease

The aetiology of nasal polyposis is still unknown [6]. Aspirin-exacerbated respiratory disease (AERD), although represented with a low incidence in the general population, is often present in patients suffering from nasal polyposis or asthmatic symptoms [7]. Since polyposis due to AERD is prone to recurrence when treated only surgically [7], AERD, characterised by the presence of the Samter triad—the presence of nasal polyposis, asthma and intolerance of acetylsalicylic acid (ASA) [8]—has to be ruled out when polyposis is present. The prevalence of AERD ranges from 0.6–5.4% and up to 15% of the patients with asthma are suspected to have ASA intolerance [9]. The correlation of nasal polyposis and ASA intolerance is still poorly understood.

AERD may emerge from ASA intolerance and is characterised by mucosal infiltration of basophils, eosinophils and mast cells, which are able to release prostaglandin [7]. In patients with AERD, a reduced expression of E-prostanoid receptors may reduce the capacity of prostaglandin E2 to mediate anti-proliferative and anti-inflammatory effects [10]. More precisely, fibroblasts derived from nasal polyps of AERD patients express lower levels of the E-prostanoid 2 receptor than fibroblasts derived from nasal mucosa of control patients [10]. Sex hormones, such as estradiol and progesterone, regulate E-prostanoid receptors and inflammation [11]. The main subtypes of the E-prostanoid receptors ERα and ERβ are expressed in various immune cells, such as lymphocytes, macrophages and dendritic cells [12]. The E-prostanoid receptors bind within the nucleus to DNA that is associated with transcription factors such as NF-κB, an important regulator of immune cell function [12].

### Chronic rhinosinusitis and sex hormones

The regulation of inflammation by sex steroids such as estradiol is of importance since it aids to explain the sex differences observed in inflammation, immunity to infection and autoimmunity. Also, sex hormones like progesterone and estradiol are involved in many different physiological and also pathological processes including respiratory health [13]. A sex preponderance in asthma [14] and ASA [15] intolerance has been observed. Hormonal fluctuations may be responsible for exacerbations of asthma in women [16] and nasal allergic reactivity [17].

The association between female sex hormones and asthma remains controversial [16]. Others state that ASA intolerance has a male predominance, although when diagnosed in women, the disease is more severe [7]. By contrast, AERD has a female predominance [18].

The sex hormones estradiol, progesterone and testosterone can modulate allergic reactions and inflammation [16,19]. Diseases associated with inflammation are aggravated during menses showing clearly sex-associated differences [20]. Estrogen, especially 17ß-estradiol (E2) may be responsible for the sex-associated differences and is well-known as an anti-inflammatory hormone [16]. In nasal polyposis, numerous mast cells express estrogen- and progesterone-receptors (ER/PR) [21]. This may suggest an interrelation between the hormonal status of the patients and nasal polyposis. Asthma-exacerbations can occur in the pre-menstrual time [16]. The pre-menstrual time describes mainly the late luteal phase at the end of cycle just prior to menstruation. Both estrogen and progesterone levels reach a peak around the mid-luteal phase and show a rapid decline in the late luteal phase thereby contributing to menstruation [22]. Women are less affected by asthmatic symptoms after their menopause [16,19], which might implicate a correlation to the levels of sex hormones, especially estradiol [8].

Eosinophils greatly influence allergic inflammation and asthma and eosinophilia are highly represented (up to 50%) in patients with ASA-sensitivities [9]. Estradiol regulates the behaviour of eosinophils, including their durability [10] since eosinophils express ER-alpha receptors [10]. During the female menstrual cycle, the amount of eosinophils fluctuates. After the menopause, when the estrogen level decreases, women are more affected by asthmatic symptoms and even previously non-affected women can develop asthma [10]. Under hormone substitution in the menopause, asthmatic symptoms can be reduced [10]. On the contrary, an exacerbation of lung disease is observed during the phase of the menstrual cycle with high estrogen levels [10]. There is also an association between type 2 inflammation of nasal polyposis and the development of asthma proposed in other studies [23]. Type 2 inflammation in the airway is based on the accumulation of different immune cells including eosinophils, mast cells and T helper 2 cells as well as a characteristic profile of cytokines (e.g., IL-4 and IL-5) [15].

The complex choreography of hormones, ASA intolerance, inflammation and immune cells in AERD is not well understood. From investigations on patients with bronchial asthma, there is increasing evidence on the role of sex and the influence of sex hormones (for review on immune cells and sex hormones, see [24]).

Hitherto, there are no studies available investigating the hormonal state in patients with nasal polyposis. In the present retrospective study, it will be therefore investigated whether patients with nasal polyposis and ASA intolerance have lowered plasma levels of estradiol, progesterone or testosterone. A possible relationship between radiological scores and varying levels of sex hormones in nasal polyposis will be also investigated.

## Material and methods

All patient data were extracted retrospectively from the medical records of the patients. This study is a retrospective analysis of clinical data. All data used in the presented study were completely anonymized and de-identified before access and analysis. All patients gave full informed consent upon their admission for their data to be used (analysed and published) for scientific purposes. In our Clinic, IRB approval is not required for retrospective anonymous analysis. Our study follows the principles of the Declaration of Helsinki. A total of 61 patients treated during the period from December 2015 to January 2018 in Hannover Medical School were included. Inclusion criteria were: age of 18 years or older, indication for first-time surgical treatment either for nasal polyposis or for septal deviation and pre-surgical blood sample analysis including hormonal state. From routine blood investigations, the levels of estradiol, progesterone and testosterone as measured in the peripheral blood of the patients were extracted retrospectively from the clinical records of the patients. All blood samples were taken prior to surgery. Hormonal concentration references were laboratory age- and sex-matched with no uniform limit for all patients. For quantification of sex hormones, the cobas e 801 module (Roche Diagnostics, Mannheim, Germany) was used. Based on the electrochemiluminescence immunoassay "ECLIA”, the Elecsys estradiol III, Elecsys progestrerone III and Elecsys testosterone II assays were used for analysis. These immunological quantitative hormone analyses were performed by the Central Laboratory of our Clinic. There was no available information on the menstrual cycle, although there was information on the menopause status and the use of birth control or hormone therapy in some but not all women. Women were included in the study even if there was no information available on their use of birth control or hormone therapy.

The septal deviation group has been added as control to include patients also suffering from mechanical obstruction of the nose due to anatomical and not pathological reasons. Each patient with nasal polyposis was tested for ASA-intolerance and received pulmonary function testing, blood testing, electrocardiography, rhinomanometry, smell test and skin-prick test. For three days, the patients followed a low salicylate diet and were treated prophylactically with pantoprazole to avoid stomach irritation. The ASA-intolerance test was performed afterwards for 3 more days with ongoing diet. Patients ingested acetylsalicylic acid starting at a concentration of 250 mg per day and increasing the concentration up to 500 mg per day–if possible. As part of the pre-operative evaluation, all patients also underwent a cone-beam computed tomography (CBCT), which in some of them was performed just prior to surgery and in others weeks to months before surgery. Based on the scans, the Lund and Mackay score [25] and the Kennedy score [26] were determined for the radiological staging of chronic rhinosinusitis.

For the Lund and Mackay score, six anatomical parts (i.e., maxillary sinus, sphenoid sinus, frontal sinus, anterior ethmoidal cells, posterior ethmoidal cells and ostiomeatal complex) on each side were analysed separately. Each part was scored from 0 to 2, judging the severity of mucosal inflammation and fluid accumulation. The ostiomeatal complex was analysed separately using score 0 (no obstruction) and 2 (obstruction). All scores summed up to a total bilateral score, ranging from 0 up to 24 points. The lower the score, the higher the lucence of the sinuses, whereas increasing scores represent a higher opacity.

The staging system by Kennedy is divided into five different stages of sinus disease ranging from 0 to 4. Stage 0 represents a normal (<2 mm) circulating mucosal thickening of sinuses. Stage 1 indicates increased mucosal thickening localised in the ethmoid sinuses only. In stage 2, the ethmoid as well as another sinus and in stage 3, two or more additional sinuses are affected in each side. For stage 4, diffuse sinunasal polyposis or an infestation of all sinuses must be present.

Statistical analysis was performed using Graph Pad Prism version 6.0. The Chi-Square test of independence was used to determine if there is a significant relationship between nasal polyposis or ASA-intolerance and hormone levels. For statistical analysis, a two-tailed t-test was used with a confidence level set at 95% and p = 0.05 or less was considered as significant. Both radiological scores were analysed statistically by using the Fisher’s exact test.

## Results

Of the total of 61 patients included in the study, 30 were male and 31 were female. The age of the patients ranged from 21–81 years for men and from 20–74 years for women (Table 1). Lower or normal/elevated levels of sex hormones in our analysis refer to the laboratory sex- and age- matched reference range and were therefore analysed separately for women and men. Although not statistically significant, mean estradiol levels were lowered in the patient group with nasal polyposis, especially in the group of ASA+ patients (Table 1). The minimum value for estradiol for men was 12.2 pg/ml and the maximum 42 pg/ml. Age-related reference values within this range were considered as normal. A few male patients (n = 4 of the group of nasal polyposis and n = 3 of the group of septal deviation) had elevated estradiol levels (Table 2, lower table). The minimum value for estradiol for women was 35 pg/ml and the maximum 375 pg/ml and reference values within this range were considered as normal. Several women, (n = 19) especially in the group of patients with nasal polyposis (n = 13)—showed estradiol levels lower than 35 pg/mL, but none of the analyzed women in both groups of patients showed estradiol levels higher than 375 pg/mL.

**Table 1:** Patient demographic data including the levels of estradiol in pg/ml.

	**Male**	**Female**	**Male**	**Female**
N; age range (median +/- SD)	N; age range (median +/- SD)	estradiol level	estradiol level (mean +/- SEM) (pg/ml)
		mean +/- SEM (pg/ml)	
**Nasal polyposis**	10; 21–66 y (50.5 +/- 14.23 y)	16*; 30–74 y (47 +/- 11.89 y)	31.15 +/- 3.88	54.51 +/- 13.74
**ASA negative**	6;21–66 y (49 +/- 6.19y)	2; 30–32 y (31 +/- 1.41 y)	32.3 +/- 5.01	109.6 +/- 86.9
**ASA positive**	3^§^; 21–66 y (51 +/- 14.23 y)	14; 30–74 (48 +/- 10.77 y)	30.38 +/- 7.04	46.64 +/- 11.31
**Septal deviation**	20; 21–81 y (39 +/- 17.219y)	15**; 20–67 y (40 +/- 14.96 y)	31.82 +/- 3.63	118.6 +/- 27.46
	N = 30	N = 31		

* 7 women under hormonal substitution for birth control and 3 after hysterectomy; unknown for the remaining 6 patients.

** 6 women under hormonal substitution for birth control and 4 without any hormonal substitution; unknown for the remaining 5 patients.

^§^ASA test not possible in one case.

**Table 2:** Number of patients with normal/elevated or low levels of estradiol.

	**Low estradiol**	**Normal/high estradiol**	**Total**	
**Nasal polyposis**	12	14	26	
**Septal deviation**	5	30	35	
	N = 17	N = 44	**N = 61**	
	Male*		Female**	
	Low estradiol	Normal/high estradiol	Low estradiol	Normal estradiol
**Nasal polyposis**	0;	10; 21–66 y (50.5 +/- 14.23 y)	12; 30–74 y (48 +/- 12.49)	4; 30–56 y (43.5 +/-10.69)
**Septal deviation**	0;	20; 21–81 y (39 +/- 17.19 y)	5; 31–67 y (60 +/- 17.23)	10; 20–54 y (38 +/- 11.47)
	N = 0	N = 30	N = 17	N = 14

* Range for normal estradiol values varied for males.

** Range for normal estradiol between 35–375 ng/ml for females.

**Fig 1:**
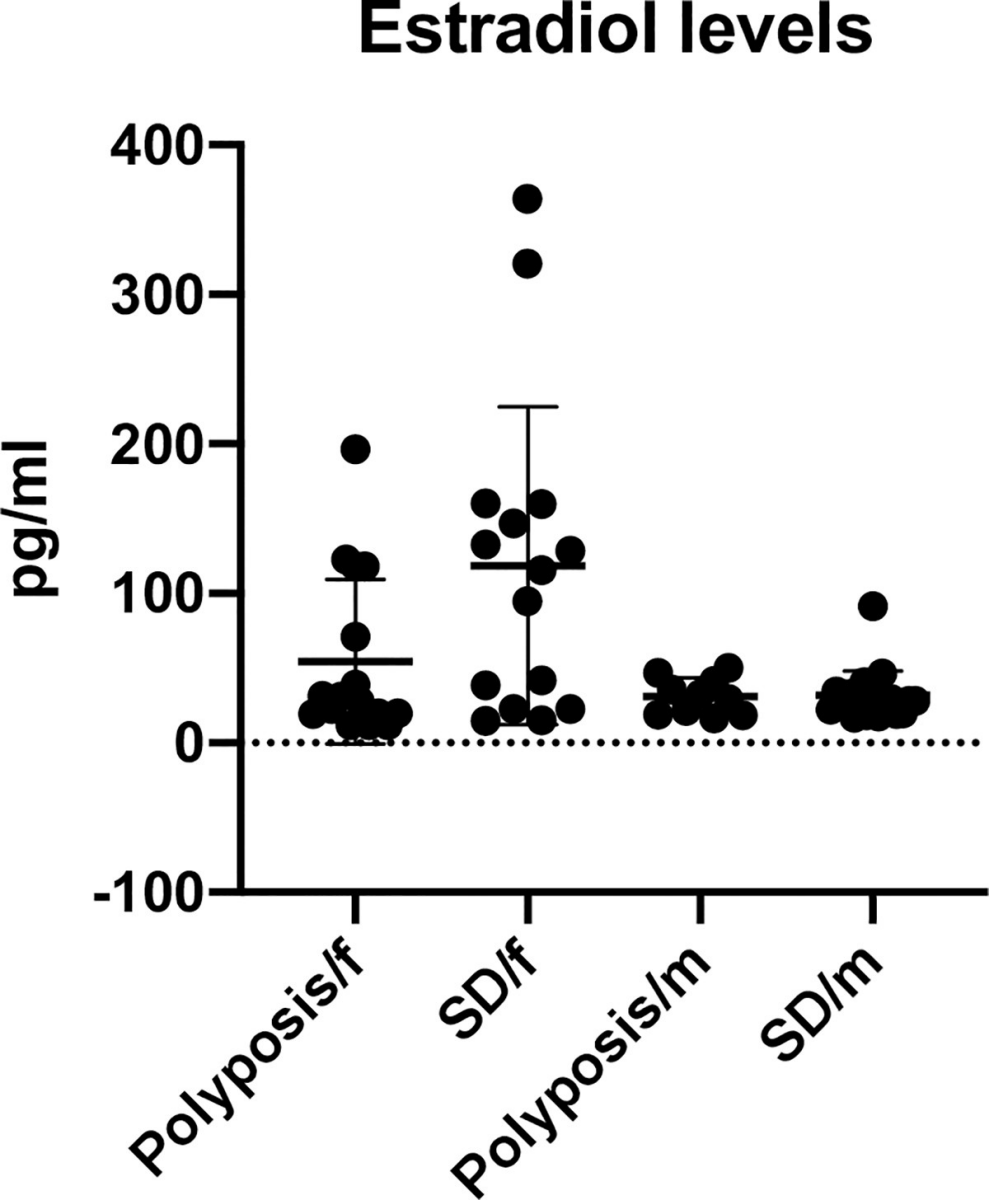
Level of estradiol in the group of patients with nasal polyposis or with septal deviation.

In the group of patients with nasal polyposis, nearly half of the patients had low (n = 12) estradiol levels (Table 2, upper table). In the group of patients with septal deviation, there were only a few patients with lowered levels of estradiol (5 out of 35) (Table 2, upper table). When looking at the sex, none of the male patients had lowered estradiol levels, but normal or elevated values were obtained for all males included (Table 2, lower table). By contrast, none of the women had elevated estradiol levels (Table 2, lower table). The estradiol levels of each individual patient are depicted separately for males and females in Fig 1.

**Fig 2:**
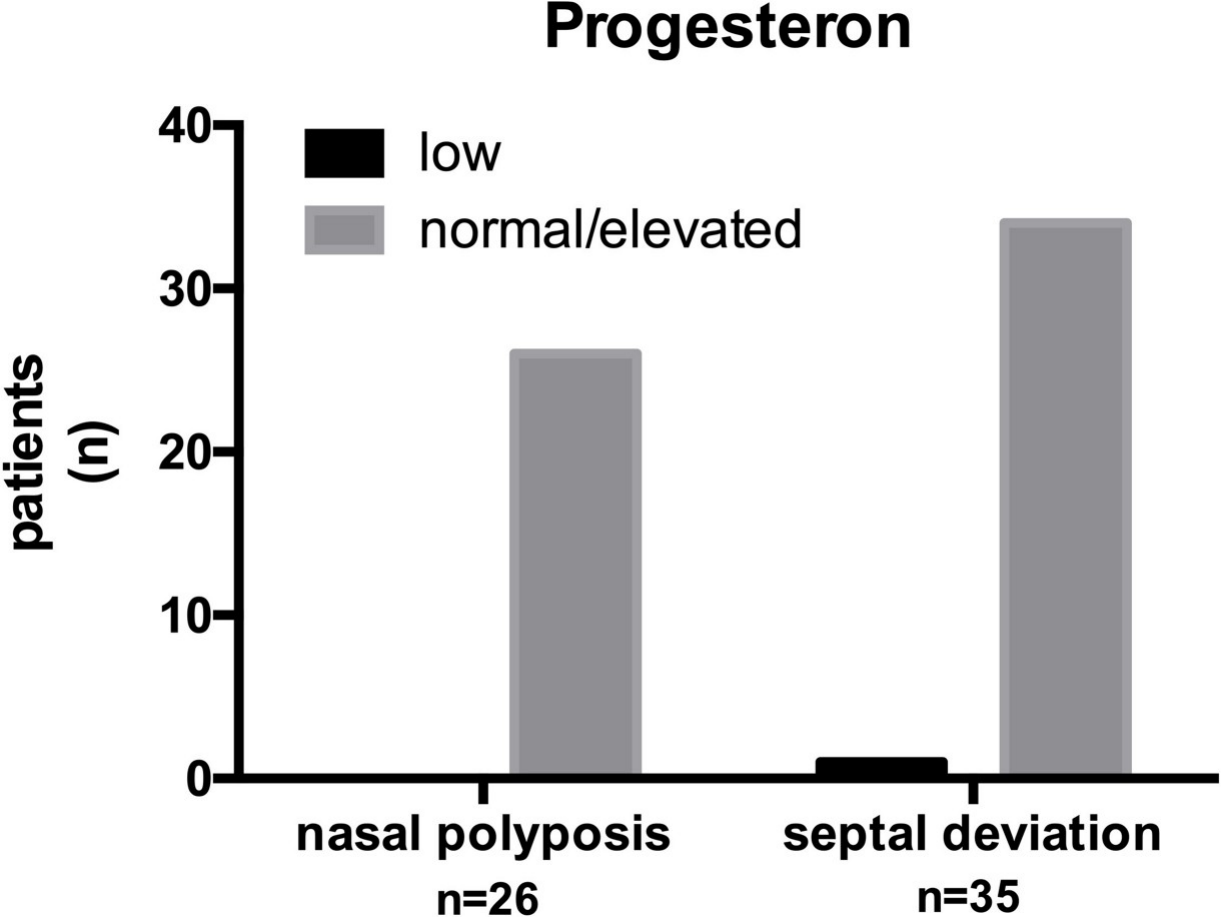
Level of progesterone in the group of patients with nasal polyposis or with septal deviation.

There was no significant difference between males and females and their risk to develop nasal polyposis. However, our results show a significant (p < 0.05) difference of estradiol levels between the two patient groups (nasal polyposis and septal deviation) χ^2^ (1, N = 61) = 2.745, p = 0.01. The odds that lowered estradiol levels correlate with the presence of nasal polyposis was 5.14 times greater (95% CI for OR, 1. 5 and 17.44) than the odds for patients with normal or elevated estradiol levels. The relative risk was 3.2. All patients with nasal polyposis (n = 26) and nearly all patients with septal deviation (n = 35) had normal or elevated levels of progesterone (Fig 2).

The majority of the patients with nasal polyposis had normal or elevated levels of testosterone (21 of 26). The group of patients with septal deviations (n = 35) showed a similar result (27 of 35). Nearly all (but one of the patients with septal deviation) patients included in the study had normal to elevated progesterone levels. Our results showed no statistically significant (p > 0.05) difference of progesterone or testosterone levels between the groups of patients with nasal polyposis and septal deviation; (progesterone χ^2^ (1, N = 61) = 0.7552, p > 0.05; testosterone χ^2^ (1, N = 61) = 0.117, p > 0.05).

Of the patients with nasal polyposis (n = 26), 25 were tested for ASA-intolerance and were included in the following analysis (Table 1). Among these, 17 were tested positive and 8 were tested negative for ASA intolerance (Table 1). Patients with lowered estradiol levels had an increased risk to develop ASA intolerance; χ^2^ (1, N = 25) = 5.940, p = 0.01. When analyzing the risk of males and females to develop ASA intolerance, 14 females and 3 males had ASA intolerance; χ^2^ (1, N = 25) = 5.940,

P = 0.01. When analyzing the risk of males and females to develop ASA intolerance, 14 females and 3 males had ASA intolerance. The two-tailed P value as calculated by Fisher’s exact test was *p = 0.01; χ^2^ (1, N = 25) = 7.767, p = 0.01. Indeed, of the 14 female patients with nasal polyposis and ASA intolerance, 11 showed reduced levels of estradiol. By contrast, none of the 3 male patients with ASA intolerance and polyposis had lowered estradiol levels.

A significant relationship between ASA-intolerance and levels of progesterone or testosterone was not detected (confidence level 95%; χ^2^ (1, N = 25) = 4.0467552, p > 0.04). The Fisher’s exact t-test was not significant for progesterone and ASA-intolerance (p > 0.99).

As expected, patients with nasal polyposis had a higher Lund and Mackay score than patients with septal deviation. There was no relationship between the scores assessed by Lund and Mackay and the sex hormone levels (Fisher’s exact t-test p > 0.65 for estradiol; p > 0.99 for progesterone and for testosterone) (Table 3). Similar results were found with the Kennedy stages (Fisher’s exact t-test p > 0.99 for estradiol and for progesterone and p = 0.37 for testosterone) (Table 4).

**Table 3:** Lund and MacKay score: Nasal polyposis (n = 25).

**Lund & Mackay score**	**Estra- diol low**	**Estra- diol normal / elevated**	**Progeste-rone low**	**Progeste-rone normal / elevated**	**Testoste-rone low**	**Testoste-rone normal / elevated**
**< 12**	3	2	0	5	1	4
**> / = 12**	8	12	0	20	4	16
**Total**	11	14	0	25	5	20

**Table 4:** Kennedy score: Nasal polyposis (n = 25).

**Kennedy score stage**	**Estradiol low**	**Estradiol normal / elevated**	**Progeste-rone low**	**Progeste-rone normal / elevated**	**Testoste-rone low**	**Testoste-rone normal / elevated**
**0**	0	0	0	0	0	0
**1**	0	0	0	0	0	0
**2**	1	1	0	2	1	1
**3**	2	5	0	7	1	6
**4**	8	8	0	16	3	13
**Total**	11	14	0	25	5	20

One imaging in the group of patients with nasal polyposis was missing.

## Discussion

The aetiology and progression of chronic inflammatory diseases is influenced by the menstrual cycle and pregnancy. For example, the menstrual cycle might affect the numbers of functional immune cells [20]. Baseline inflammation and immune cell activation might explain the menstrual-associated fluctuations described in chronic and acute diseases [27]. Specifically autoimmune diseases, asthma, diabetes and cardiac diseases display worsening of symptoms premenstrually or during menses [20,27]. Indeed, the results from the herein presented study demonstrate a correlation between lowered estradiol levels and nasal polyposis. From the patients with ASA-intolerance in our study, two-third showed reduced estradiol levels. All of these patients were female and showed also a significant relationship between the levels of estradiol and ASA-intolerance. Thus, patients with lower levels of estradiol and females seem to be at an increased risk to develop ASA intolerance.

The sex hormone estradiol is involved in chronic diseases, for example asthma [17]. Estradiol exerts anti-inflammatory effects by suppressing the immune system [17]: Due to the inhibition of TNF-alpha, interferon-γ and natural killer cells, cellular apoptosis is prevented [16]. Hormone supplementation may therefore be one option to alleviate the symptoms of diverse chronic diseases, including nasal polyposis. Due to its versatile functions on a cellular and molecular level, more studies are needed to effectively utilise estradiol for targeted therapies for the treatment of patients with ASA intolerance and nasal polyposis.

Other sex hormones could also be involved in the pathogenesis of chronic inflammatory disease. Progesterone was shown to modulate allergic reactions [11] and testosterone to decrease airway inflammation [17]. In patients with nasal polyposis or ASA intolerance, however, there was no difference in the levels of progesterone and testosterone between the groups.

The risk for developing nasal polyposis and ASA intolerance might be different between males and females due to differences in hormonal fluctuations. The groups of patients in this study showed a similar distribution of sex and age (Table 1). More women than men were found in the group of patients with nasal polyposis, most of them younger than 50 years. With regard to the common known mean age of 45–50 years in nasal polyposis [28,29], the mean age of our patients was within this range (47.5 years for males and 47.38 years for females). Interestingly, this is close to the typical age of menopause.

A complex network of processes in the formation of chronic rhinosinusitis with polyposis has been suggested recently involving gross epithelial damage and repair reactions, eosinophil and macrophage cell infiltration, and tissue remodelling [30]. These changes are induced by IL-25 via the activation of MAPKs and NF-κB signalling pathways [31] and estradiol is known to attenuate MAPK activity [32]. Chemosensory cells of the nasal epithelium are the source of IL-25 and are increased in the inflamed nasal epithelium of patients with nasal polyposis [33].

Radiological scores have been introduced to scale the severity of CRS and nasal polyposis. The most widely accepted scores are based on Lund and Mackay [34,35] and on Kennedy [26]. There was no significant relationship between lowered levels of sex hormones and higher scores as assessed according to Lund and Mackay or to Kennedy. The general population seems also to not have a Lund and Mackay score of zero [34], which has to be recognised in our study. Whether the Lund and Mackay or Kennedy scores might not be sensitive enough or whether the sex hormone levels may correlate with the onset and recurrence but not with the severity of the disease needs to be subjected to further investigation. Thus, a greater number of patients is needed to explore a possible relationship between lowered levels of sex hormones and radiologic grading of disease severity thereby excluding the variations in the general population.

In conclusion, our study shows that sex hormones may play a role in the pathogenesis of nasal polyposis. Patients with nasal polyposis have lower estradiol levels than patients with septal deviations. Despite the finding that low levels of estradiol correspond to a higher incidence of nasal polyps, there was no significant relationship to radiologic grading of disease severity (Tables 3 and 4). Our results show higher CT scores / stages (according to Lund and Mackay or to Kennedy) in patients with nasal polyposis when compared to patients with septal deviation. Based on our results, however, no significant relationship of CT scores with lowered levels of sex hormones was found.

Our results lead to the hypothesis that especially females with lower estradiol are at a higher risk to develop nasal polyposis and ASA. Whether these factors are associated with the severity of chronic rhinosinusitis needs further assessment. Other factors associated with the presence and severity of CRS, nasal polyposis and ASA intolerance such as the use of birth control or hormonal therapy, number of childbirths, other chronic diseases and medication to treat them, obesity, smoking, seasonal fluctuations with regard to nasal allergic reactions and asthmatic symptoms could not be assessed sufficiently in our retrospective analysis and need further investigation. If the significant relationship between lowered estradiol levels and nasal polyposis found in our study could be verified in future studies involving a larger number of patients, it introduces unexplored opportunities of a targeted molecular therapy for nasal polyposis.

Possible limitations in our study are the small number of patients that have been included. More studies are required to verify our hypothesis that reduced estradiol levels may be associated with ASA intolerance in patients with nasal polyposis. Other personal and environmental factors, which were not considered in our study, could cause the tendency and have to be further explored in future studies with a greater number of patients and with focus on additional characteristics. Genotype-specific variants of chronic rhinosinusitis are currently emerging by genome wide linkage and association studies [36] and may also aid to identify sex-specific differences. It may also be interesting to further explore the influence of CRS on cognition in future studies. Fatigue, slowed thinking as well as struggle with concentration and memory were reported by patients with CRS in other studies [2]. Objective tests showed worsening of for example simple reaction time and mathematical processing in patients with CRS [2]. Knowledge about potential risk or protective factors for severe cognitive dysfunction may also be important for the choice of therapy and is also relevant for the quality of life of patients. One main aspect that also needs consideration is the selection of patients. In our study, patients were not selected by potential risk factors and the risk to develop nasal polyps due to the presence of other variables cannot be judged. An additional limitation of our study is the wide age range of the included patients. Future studies analysing different groups of ages in detail are needed to specify factors playing a role in the development, severity and therapy of ASA intolerance, nasal polyposis and their relationship to the level of sex hormones. Our results concerning a correlation between varying levels of sex hormones and the Lund and Mackay score might be influenced by normal distribution in population. A greater number of patients might compensate the normal distribution found in the healthy general population. In addition, the time point of the blood investigation in relation to the CBCT scans as well as in relation to the menstrual cycles (where it applies) are further variables that need to be controlled in specifically designed clinical trials.

The results of our study show a significant relationship between lowered levels of estradiol and nasal polyposis. Further analyses are required in order to evaluate if hormonal substitutions in patients with lowered estradiol levels and nasal polyposis could prevent recurrence of disease after surgery. Although we did not find any other significant relationships in our analysis, we suggest different factors that should be further explored in future studies to develop an individual and most effective targeted therapy for patients with ASA-intolerance and nasal polyposis.
